# Movement-Provoked Paroxysmal Neuralgia Following Longitudinally Extensive Transverse Myelitis in Neuromyelitis Optica Spectrum Disorder: A Presumed Ephaptic Spinal Cord Phenomenon

**DOI:** 10.7759/cureus.113809

**Published:** 2026-08-02

**Authors:** Angela Philips, Johan Sosa De La Cruz, Robert Ungerer, Prudvi Tarun Betha

**Affiliations:** 1 Neurology, University of Illinois College of Medicine Peoria, Peoria, USA; 2 Internal Medicine, University of Illinois College of Medicine Peoria, Peoria, USA

**Keywords:** ephaptic transmission, letms, mogad, paroxysmal pain syndrome, spinal cord phenomenon

## Abstract

Neuromyelitis optica spectrum disorder (NMOSD) is an autoimmune astrocytopathy frequently complicated by neuropathic pain. Paroxysmal symptoms likely arise from ephaptic transmission within demyelinated spinal cord tracts and are underdescribed when presenting as isolated sensory phenomena, unlike the well-characterized tonic spasms.

We report a 76-year-old woman with aquaporin-4 (AQP4) antibody-positive NMOSD recovering from her first disease flare, which initially manifested as longitudinally extensive transverse myelitis (LETM). Weeks later, she developed sudden, excruciating, movement-provoked paroxysmal burning pain in the lower extremities. Episodes lasted 4-8 seconds and occurred approximately 20 times daily. Serial neurologic examinations revealed no new deficits suggestive of relapse, and repeat MRI was deferred as relapse was considered clinically unlikely. Symptoms were refractory to gabapentin but improved dramatically within 24 hours of initiating oxcarbazepine 300 mg twice daily.

The clinical phenotype and rapid response to sodium channel blockade support a sodium channel-mediated mechanism, most likely ephaptic transmission, rather than trigger-independent ectopic firing, which remains sparsely described in the literature. Recognition of this phenomenon is crucial to avoid misclassification as relapse, unnecessary imaging, and unwarranted escalation of immunotherapy.

## Introduction

Neuromyelitis optica spectrum disorder (NMOSD) is a severe inflammatory demyelinating disorder of the central nervous system characterized by optic neuritis and longitudinally extensive transverse myelitis (LETM), associated with aquaporin-4 (AQP4) antibodies [1,2]. Pain is highly prevalent in NMOSD, affecting up to 80-90% of patients, and may be chronic, central neuropathic, or radicular in character [3,4].

Paroxysmal symptoms, including tonic spasms, dysesthesias, and brief sensory phenomena, are well recognized in demyelinating disorders. Painful tonic spasms are the best-described paroxysmal manifestation in NMOSD, whereas isolated movement-provoked sensory paroxysms have been reported much less frequently. These symptoms are commonly attributed to ephaptic transmission between partially demyelinated axons. Other proposed mechanisms include ectopic spontaneous firing resulting from sodium channel redistribution and membrane potential oscillations [5], as well as axonal hyperexcitability due to altered ionic conductance [5-7]. Unlike ephaptic transmission, which requires activation of neighboring axons and therefore may be precipitated by movement or other stimuli, ectopic spontaneous firing can occur without an external trigger.

Ephaptic transmission refers to abnormal electrical cross-talk between adjacent demyelinated axons, enabling lateral propagation of action potentials without synaptic mediation. Although paroxysmal tonic spasms in NMOSD are well recognized and typically respond to sodium channel blockers such as carbamazepine and oxcarbazepine, isolated movement-provoked sensory paroxysms remain considerably less well described [8].

This case highlights the importance of recognizing isolated movement-induced sensory paroxysms as a distinct, sodium channel-mediated phenomenon in patients recovering from an NMOSD attack, as new paroxysmal symptoms may be mistaken for disease relapse. Distinguishing this mechanism from relapse-related pathology and from other paroxysmal phenomena such as ectopic firing may help clinicians select appropriate pharmacologic treatment while avoiding unwarranted diagnostic and therapeutic interventions.

## Case presentation

A 76-year-old woman with recently diagnosed NMOSD presented in January 2026 with several days of severe episodic lower extremity pain. Her initial flare two months before admission had manifested as LETM and was treated with high-dose intravenous corticosteroids followed by plasma exchange. At the time of presentation, she was receiving a prednisone taper. The paroxysmal pain began approximately eight weeks after the initial LETM episode while she was recovering from the acute attack.

She described sudden, excruciating burning and shooting pain that began in the right foot and ascended proximally toward the lower back. Each episode lasted 4-8 seconds, was consistently triggered by leg movement, and occurred approximately 20 times per day. Although initially confined to the right leg, intermittent involvement of the left lower extremity developed. Pain intensity was 10/10, significantly impairing mobility and activities of daily living. She denied tonic spasms, new weakness, altered consciousness, or bowel and bladder changes. Escalating doses of gabapentin had provided no meaningful relief.

Vital signs and general examination were unremarkable. Neurologic examination showed an alert, fully oriented patient with intact cranial nerves. Strength, according to the Medical Research Council (MRC) scale, was 5/5 throughout, except for mild right hip flexion and extension weakness (4+/5), unchanged from prior examinations. Reflexes were brisk in both lower extremities (3+ patellar and Achilles), plantar responses were flexor, and vibration and proprioception were decreased in the right leg. Light touch and pinprick were intact. Serial examinations revealed no new motor deficits, no new sensory level, and no clinical evidence of relapse.

The differential diagnosis included central neuropathic pain related to prior LETM, radiculopathy, Lhermitte phenomenon, NMOSD relapse, and a paroxysmal sensory phenomenon due to ephaptic transmission. The brief, stereotyped, movement-triggered nature of the attacks in the absence of new neurological deficits argued strongly against relapse. Radiculopathy was considered unlikely given the absence of dermatomal distribution, persistent pain, or lower motor neuron findings. Lhermitte phenomenon was also considered less likely because episodes were triggered by leg movement rather than neck flexion and were confined to the lower extremities.

**Figure 1:**
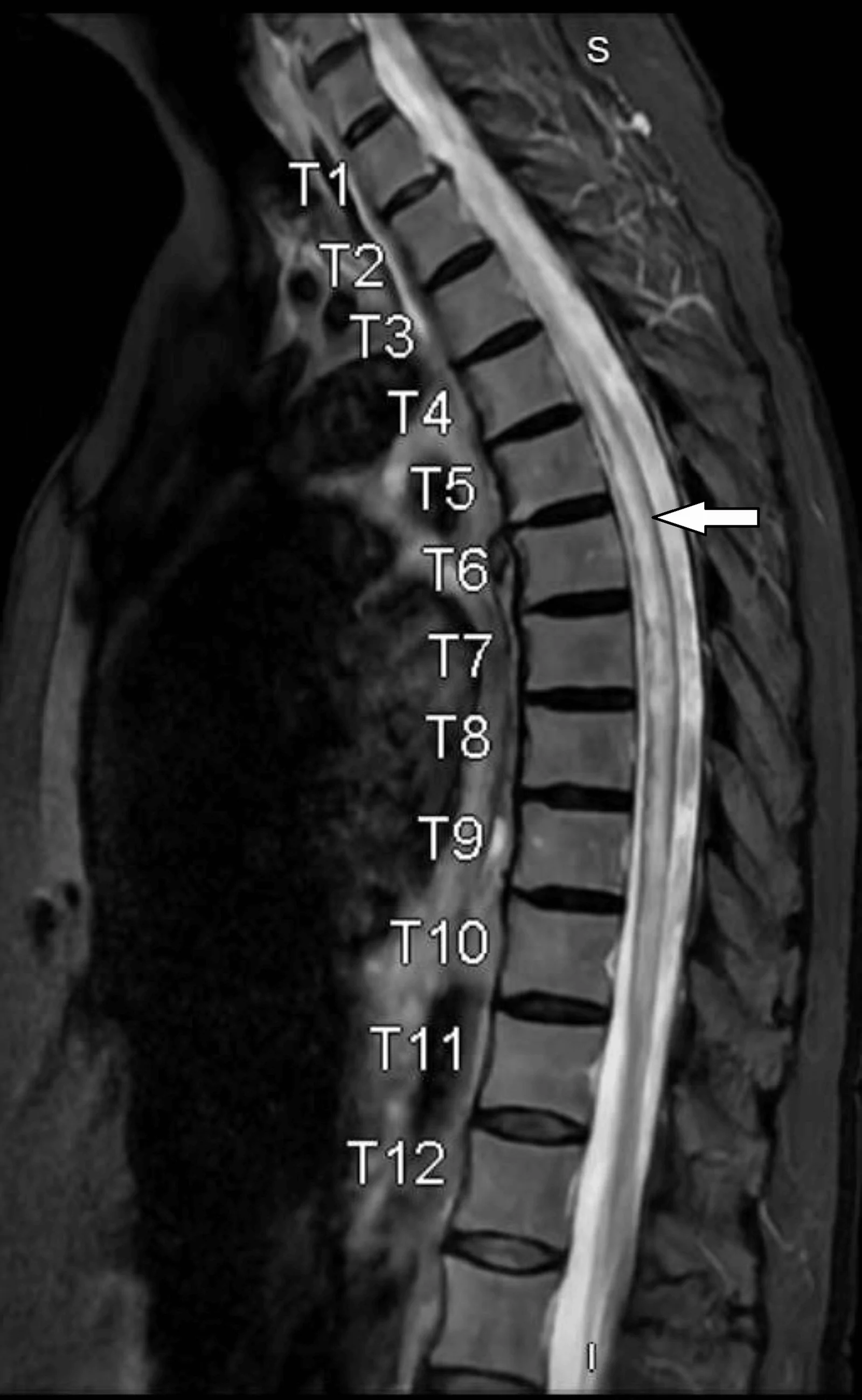
MRI T2-STIR showing extensive T2 signal abnormalities involving the central cord spanning T2-T11, with signal abnormalities spanning T4-T9.

The clinical picture was most consistent with a movement-provoked paroxysmal sensory phenomenon arising from previously demyelinated spinal cord pathways. Loss of myelin permits abnormal electrical coupling between adjacent axons, allowing minor mechanical activation to trigger synchronized discharges and brief shock-like pain episodes. Repeat spinal MRI was initially considered but ultimately deferred because serial neurological examinations remained stable and an NMOSD relapse was considered clinically unlikely. Thoracic spine T2-weighted short tau inversion recovery (T2-STIR) MRI, obtained two months before the onset of paroxysmal pain during the patient's initial LETM presentation, showed extensive T2 signal abnormalities involving the central cord spanning T2-T11, with more focal signal abnormalities spanning T4-T9 demonstrating a longitudinally extensive hyperintense intramedullary lesion. Arrowheads identify the longitudinal spinal cord lesion (Figure 1).

Oxcarbazepine 300 mg twice daily was initiated. Within 24 hours, paroxysms decreased from approximately 20 per day to only two episodes, pain intensity markedly diminished, and functional mobility improved significantly. Gabapentin was tapered and discontinued. She continued her prednisone taper and was discharged home in improved condition. Follow-up in the clinic three months after the presentation showed improvement in her sensory symptoms, and the oxcarbazepine dose was further increased at nighttime due to a positive response with daytime symptoms.

## Discussion

This case illustrates a presumed ephaptic spinal cord phenomenon presenting as movement-provoked paroxysmal neuralgia following LETM in NMOSD. Ephaptic transmission occurs when demyelination exposes axonal membranes, increases sodium channel density, and reduces electrical insulation between adjacent fibers [6]. This permits lateral spread of depolarization, generating stimulus-triggered discharges between neighboring axons. By contrast, ectopic spontaneous firing arises from intrinsic hyperexcitability of injured axons and can occur without an external trigger. The consistent precipitation of attacks by leg movement in our patient therefore favors ephaptic transmission over isolated ectopic firing, although these mechanisms are not mutually exclusive and cannot be distinguished definitively in vivo [6-9].

Painful tonic spasms are well-recognized paroxysmal manifestations of NMOSD and have also been described in multiple sclerosis and, less commonly, myelin oligodendrocyte glycoprotein antibody-associated disease (MOGAD) [10]. In contrast, to our knowledge, isolated movement-provoked sensory paroxysms without accompanying tonic posturing have been reported far less frequently, making the present case an uncommon clinical presentation.

In NMOSD, spinal cord lesions are often longitudinally extensive and destructive, potentially predisposing to persistent axonal hyperexcitability during recovery [2,4]. Although the temporal relationship to LETM, stereotyped movement-provoked attacks, and rapid response to oxcarbazepine strongly support a sodium channel-mediated process, the diagnosis of ephaptic transmission remains inferential because no direct physiological test exists to confirm this mechanism in vivo.

Gabapentin binds the α2δ subunit of voltage-gated calcium channels and is primarily effective in reducing excitatory neurotransmitter release in chronic neuropathic pain. In contrast, oxcarbazepine stabilizes the inactivated state of voltage-gated sodium channels, suppressing repetitive firing and abnormal impulse propagation in hyperexcitable demyelinated axons. The marked difference in therapeutic response suggests that sodium channel-mediated hyperexcitability, rather than calcium channel-mediated neurotransmission, predominated in this patient [7,8].

The dramatic improvement within 24 hours of oxcarbazepine initiation further supports a sodium channel-mediated mechanism. Recognition of this uncommon presentation is important because misclassification as NMOSD relapse may lead to unnecessary repeat imaging or escalation of immunotherapy. Early recognition allows prompt initiation of mechanism-directed therapy with substantial symptomatic improvement.

## Conclusions

Movement-provoked paroxysmal neuralgia following LETM in NMOSD may represent a presumed ephaptic phenomenon within demyelinated spinal cord pathways. The rapid response to oxcarbazepine supports sodium channel-mediated axonal hyperexcitability, with ephaptic transmission representing the most likely, although unconfirmed, mechanism. Recognition of this uncommon presentation may help clinicians distinguish it from disease relapse, avoid unnecessary diagnostic investigations or immunotherapy escalation, and initiate targeted symptomatic treatment promptly.

## References

[1] Wingerchuk DM, Lennon VA, Lucchinetti CF, Pittock SJ, Weinshenker BG (2007). The spectrum of neuromyelitis optica. Lancet Neurol.

[2] Weinshenker BG, Wingerchuk DM (2017). Neuromyelitis spectrum disorders. Mayo Clin Proc.

[3] Qian P, Lancia S, Alvarez E, Klawiter EC, Cross AH, Naismith RT (2012). Association of neuromyelitis optica with severe and intractable pain. Arch Neurol.

[4] Bradl M, Lassmann H (2014). Experimental models of neuromyelitis optica. Brain Pathol.

[5] Kapoor R, Li YG, Smith KJ (1997). Slow sodium-dependent potential oscillations contribute to ectopic firing in mammalian demyelinated axons. Brain.

[6] Waxman SG (2026). Mechanisms of disease: sodium channels and neuroprotection in multiple sclerosis-current status. Nat Clin Pract Neurol.

[7] Matthews WB (1975). Paroxysmal symptoms in multiple sclerosis. J Neurol Neurosurg Psychiatry.

[8] Cruccu G, Di Stefano G, Truini A (2020). Trigeminal neuralgia. N Engl J Med.

[9] Kim SM, Go MJ, Sung JJ, Park KS, Lee KW (2012). Painful tonic spasm in neuromyelitis optica: incidence, diagnostic utility, and clinical characteristics. Arch Neurol.

[10] Asseyer S, Cooper G, Paul F (2020). Pain in NMOSD and MOGAD: A Systematic Literature Review of Pathophysiology, Symptoms, and Current Treatment Strategies. Front Neurol.

